# An update on the human and animal enteric pathogen *Clostridium perfringens*

**DOI:** 10.1038/s41426-018-0144-8

**Published:** 2018-08-06

**Authors:** Raymond Kiu, Lindsay J. Hall

**Affiliations:** 1 Gut Microbes and Health Programme, Quadram Institute Bioscience, Norwich Research Park, Norwich, UK; 2 Norwich Medical School, University of East Anglia, Norwich Research Park, Norwich, UK

## Abstract

*Clostridium perfringens*, a rapid-growing pathogen known to secrete an arsenal of >20 virulent toxins, has been associated with intestinal diseases in both animals and humans throughout the past century. Recent advances in genomic analysis and experimental systems make it timely to re-visit this clinically and veterinary important pathogen. This Review will summarise our understanding of the genomics and virulence-linked factors, including antimicrobial potentials and secreted toxins of this gut pathogen, and then its up-to-date clinical epidemiology and biological role in the pathogenesis of several important human and animal-associated intestinal diseases, including pre-term necrotising enterocolitis. Finally, we highlight some of the important unresolved questions in relation to *C. perfringens*-mediated infections, and implications for future research directions.

## Introduction

*Clostridium perfringens* (formerly known as *Bacillus aerogenes capsulatus, Bacillus perfringens, Bacillus welchii* or *Clostridium welchii*) is a Gram positive, spore-forming, anaerobic, rod-shaped bacterium^1^. It was first isolated and identified as a novel bacterium in 1891 by William H. Welch from the autopsy of a 38-year-old man, where gas bubbles were observed within infected blood vessels. This gas-forming trait (the original name *Bacillus aerogenes capsulatus*, ‘aerogenes’ literally means ‘air-producing’ in Latin) was later linked to gas gangrene symptoms seen in British and French soldiers during World War I^2^.

*C. perfringens* is associated with diverse environments including soils, food, sewage, and as a member of the gastrointestinal (GI) tract microbial community (i.e., microbiota) of both diseased, and non-diseased humans and animals. Notably it has been associated with humans for thousands of years as evidenced by the recent identification of *C. perfringens* using next generation sequencing (NGS) technology in the mummified GI tract of a >5000-year-old Neolithic ‘Tyrolean Iceman’ (also known as Ötzi), found in an Alpine glacier in 1991^3^.

Clinically, *C. perfringens* has been constantly associated with various significant systemic and enteric diseases, in both humans and animals, including gas gangrene (Clostridial myonecrosis), food poisoning and non-foodborne diarrhoea, and enterocolitis^4,5^. Importantly, *C. perfringens* strains are known to secrete >20 identified toxins or enzymes that could potentially be the principal virulence factors involved in pathophysiology^6^.

In this Review, we explore phenotypic and genomic features of this important and re-emerging pathogen, including virulence factors, and antimicrobial resistance (AMR) profiles, and the clinical impact of this bacterium in relation to numerous medically important intestinal diseases, across several host species. Finally, we highlight some of the important unresolved questions in relation to *C. perfringens*-associated infections, and implications for future research directions.

## Isolation, identification and typing methods

There are numerous methods for isolation of *C. perfringens*, and biochemical and molecular methods for identification as detailed in Table 1.

**Table 1:** Common isolation, identification and typing methods used in *C. perfringens* research

Method	Method details in brief	Refs
Isolation		
Direct plating	Direct plating on TSC-EYA + 18–24 h anaerobic incubation at 37 °C. Pitch black colonies with opaque halos are presumptively *C. perfringens*.	^146^
Ethanol pre-treatment	Ethanol pre-treatment (50% ethanol) for 30 mins + plating on Fastidious Anaerobe Agar (sometimes supplemented with 0.1% taurocholate). Colonies that exhibit beta-haemolysis are preliminarily identified as *C. perfringens*.	^147^
Biochemical identification		
Nagler’s reaction	Known as lecithinase (alpha-toxin) test. Egg-yolk agar plates are split into two halves, where one half contains anti-alpha-toxin, and following anaerobic incubation, positivity is defined as turbidity around colonies on the anti-alpha-toxin-free side which confirms *C. perfringens*.	^148^
Reverse CAMP test	*Streptococcus agalactiae* is streaked on blood agar, and *C. perfringens* streaked perpendicular to *S. agalactiae*. After 24–48 h of anaerobic incubation, a ‘bow-tie’ zone will form due to the synergistic haemolysis, this confirms *C. perfringens*.	^149^
Molecular identification		
16S ribosomal RNA PCR	Appropriating full-length 16S rRNA universal primers/smaller region of 16S rRNA gene in PCR to amplify 16S rRNA gene + sequencing, and identify using informatics approach (based on sequence similarity >97% to assign taxonomy).	^30^
MALDI-TOF Mass Spectrometry	Rapid and inexpensive identification method based upon the mass spectrum analysis of highly-conserved ribosomal proteins from whole bacterial cells—apply bacterial colonies straight onto the MALDI-TOF metal target and followed by appropriate treatments and analysis.	^4^
Typing		
Multiplex PCR	Multiplex PCR approach is commonly used to amplify key toxins genes to classify *C. perfringens* into 7 (A to G) different toxinotypes according to the toxin genes combination.	^150^
Genome-wide sequence search	Genome-wide search on relevant toxin genes using sequence similarity search program e.g. BLAST for toxinotyping on *C. perfringens* genomes.	^10^

**Fig. 1:**
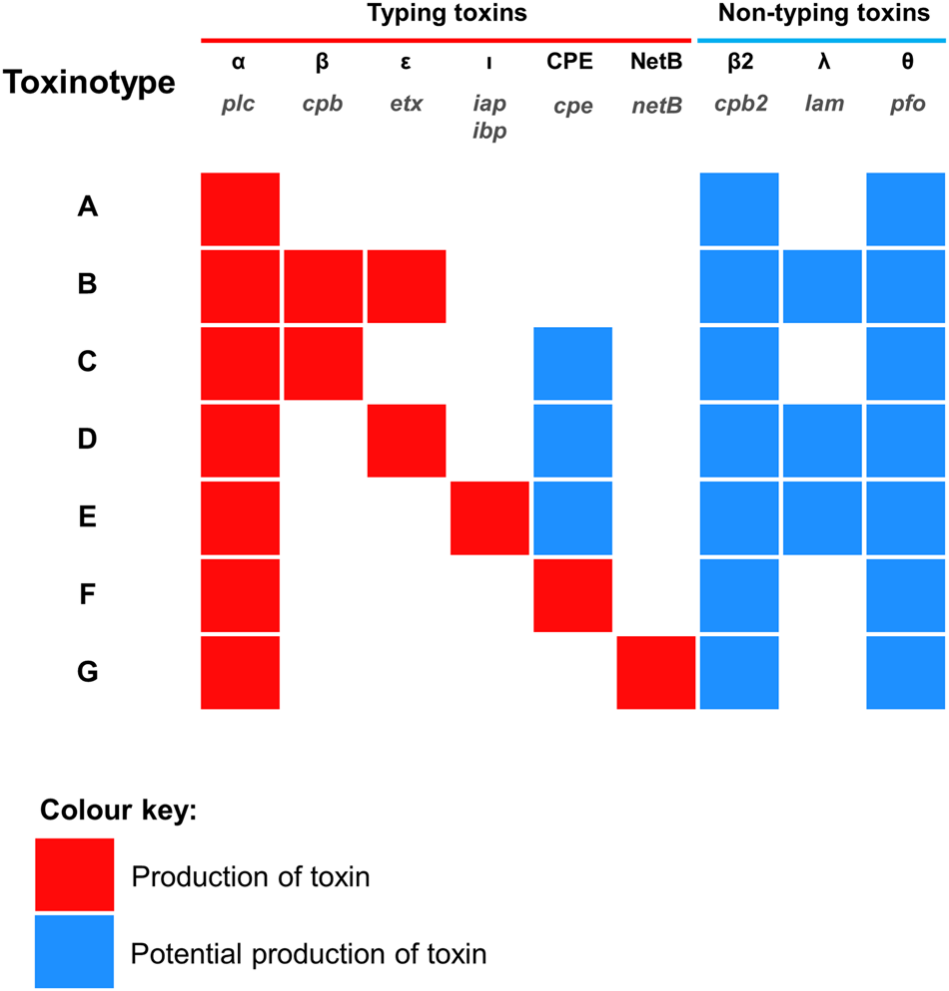
*C. perfringens* current toxinotyping system. The names of toxin genes are printed in grey

As *C. perfringens* is a pathogen, typing methods are currently used to differentiate between strains that may be associated with serious infection (Table 1). *C. perfringens* strains, clinically well-known for toxin production, are typed (recently updated for seven toxinotypes: A–G; previously only types A–E^7^) according to the combination of typing toxins (Fig. 1) they produce, i.e., α-toxin, β-toxin, ε-toxin and ι-toxin, enterotoxin (CPE) and NetB^8^. Historically this method indicated that certain toxins were associated with certain hosts and diseases e.g., type B (particularly the β-toxin it harbours), is exclusively linked to dysentery in sheep, and rarely seen in other hosts^9^. Food-poisoning associated CPE was genotyped typically in type F strains (previously named as CPE-positive type A; not to be confused with heat-resistant type C strains) although CPE can also be produced by certain type C, D and E strains, whereas β2-toxin and θ-toxin (perfringolysin O; PFO) could be found in any toxinotypes^1,10,11^. However, it is important to note that no single strain is known to produce the entire panoply of toxins^10^.

## Genomic insights

*C. perfringens* has a relatively low GC content, between 27–28%, and genome sizes range from 3.0–4.1 Mb, with 2500–3600 predicted genes present in each individual genome^10^. The first ever complete genome sequence of *C. perfringens* (strain 13) was published in early 2002; a historical gas gangrene-associated strain from 1939 (this also represents the first Gram-positive anaerobic pathogen to be sequenced)^12^. Virulence-linked genes within the sequence data include phospholipase C (α-toxin), hyaluronidase (µ-toxin), collagenase (κ-toxin), PFO (θ-toxin), and β2 toxin. Moreover, 61 sporulation-related and germination-related genes were encoded, which supports the fact that *C. perfringens* is a spore-former. Notably, most virulence genes in this type A strain were not found on genomic islands, or near insertion/transposase sequences, and GC content of those genes were similar to the average GC percentage, implying that horizontal gene transfer (HGT) of these toxin genes was an ancient evolutionary event. This study also highlighted differences in environmental adaption genes, including glycolytic enzymes, and sporulation-linked genes, whereas recent studies indicated that phylogenetic relationships between *C. perfringens* (based on core genome analysis) does not relate to clone origin or toxinotypes^1,10^

**Fig. 2:**
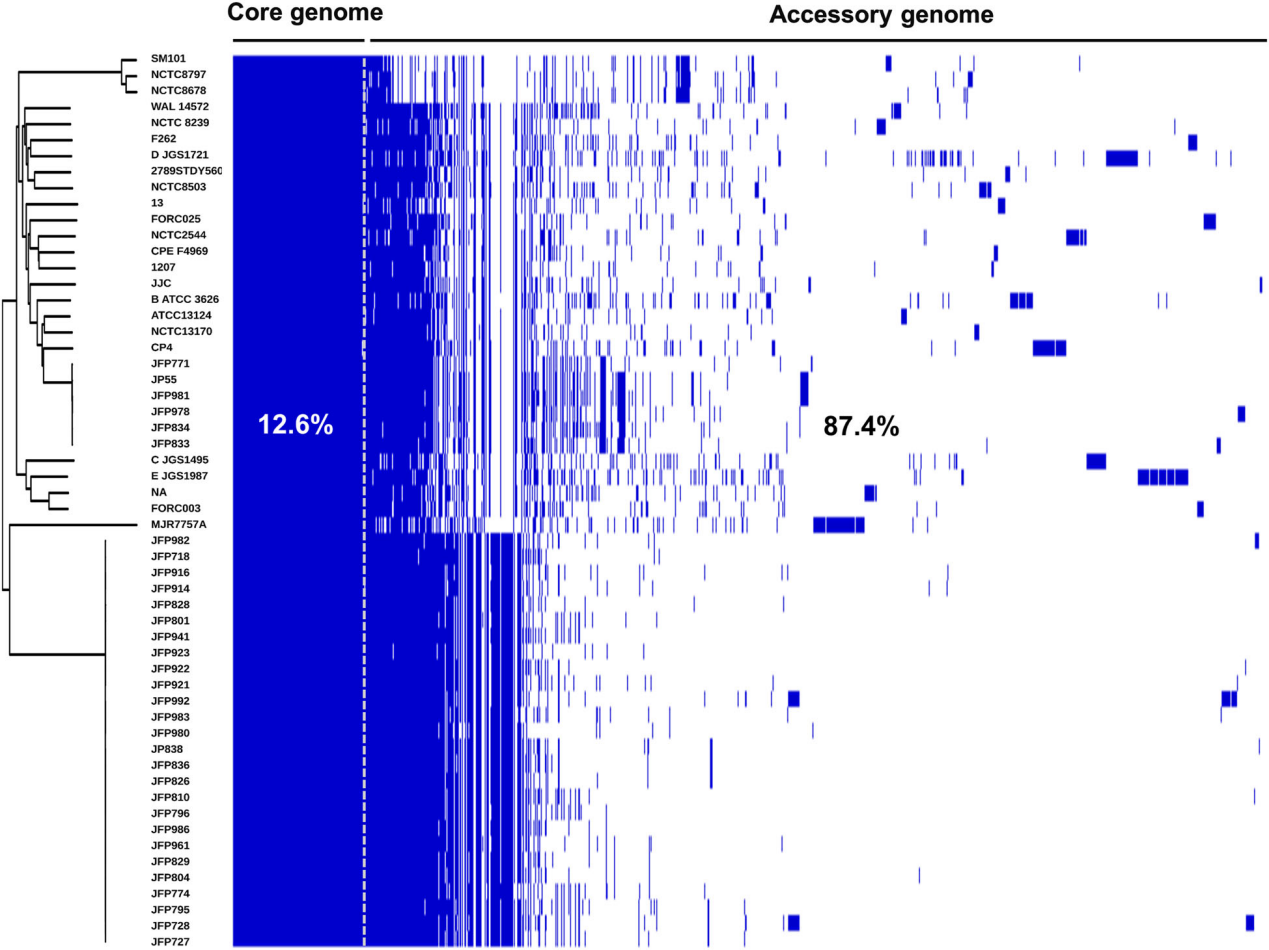
The linearised pangenome of 56 *C. perfringens* strains reveals significant genetic diversity. Each coloured block represents synteny (homolog: identical gene with >95% sequence similarity) in the pangenome. Figure adapted from Kiu et al.^10^

A recent large-scale genomic study that investigated 56 strains *C. perfringens* strains, representing the largest *C. perfringens* genomic study to date, revealed a diverse pangenome (a repertoire of genes in a defined number of genomes), with only 12.6% core genes (common genes that are present in each genome) (Fig. 2)^10^. A wide array of toxin genes was profiled in this study: α-toxin (*plc*), α-clostripain (*ccp*) and microbial collagenase (*colA*) were conserved in all examined genomes, the pore-forming toxin PFO gene *pfo* was found to be encoded in most genomes (>90%), whereas the food-poisoning causative toxin CPE gene *cpe* was consistently detected in historical food poisoning isolates (isolated before 1950s). Importantly, prevalence of aminoglycoside-resistant/anti-defensin gene *mprF* (100% detection), and tetracycline-resistant efflux protein genes *tetA (P)* (>75% detection) encoded within the genomes underlined the potential antimicrobial resistant threat of *C. perfringens*-associated infection. This study also suggested that these diverse genetic variations may have been driven by HGT, especially prophage insertion, within the ‘CRISPR-free’ genomes (no single CRISPR prophage defence system detected in >70% of the genomes).

The substantial genetic divergence in the *C. perfringens* pangenome suggests there may be additional novel virulence-related genes encoded within the ‘accessory genome’, in addition to the plasmid-borne toxins, known to be primarily responsible for specific disease pathologies. Thus, it is important to analyse plasmid-encoded genes/toxins, in tandem to chromosomally-encoded genes in the accessory genome, to provide a more complete genomic picture of *C. perfringens*. Potentially, with the aid of bioinformatics tools, more novel toxins or virulence genes will be discovered and identified for investigation in relation to *C. perfringens* pathogenicity, and disease transmission tracking in hospital and environmental settings, as has been performed for the related gut pathogen *C. difficile*^13^. To fully determine and research the pangenome, and discover potentially novel virulence genes, more isolate sequencing will be required, including addition of isolates from a range of hosts and environmental conditions, which may pave the way for translational research into new treatment strategies against *C. perfringens* infections.

## Plasmids

Importantly, *C. perfringens* strains are known to carry plasmids, which often encode virulence-associated proteins including toxins and antimicrobials^14^. Disease-associated toxins including; CPE, ε-toxin, ι-toxin, NetB, β2-toxin and binary enterotoxin (BEC) have all been detected on *C. perfringens* plasmids^10,15^. HGT of plasmids between strains is reported to be via the *tcp* conjugation locus that exists in most plasmids^16^. Important toxin-carrying plasmids will be briefly reviewed in order of toxinotypes. For comprehensive reviews on *C. perfringens* plasmids, and other related insertion sequences see^14,15^.

CPE is known to be involved in food poisoning and non-foodborne gastroenteritis. It was reported that up to 70% of food poisoning cases, causative *C. perfringens* strains (mainly type F, previously known as CPE-positive type A) were shown to encode chromosomal-*cpe*, rather than plasmid-*cpe*, while the latter was associated with non-foodborne gastroenteritis (responsible for 5–15% of all cases)^17^. Some *cpe*-associated plasmids have also been shown to encode *cpb2* genes or *iab/ibp* genes (e.g., pCPF5603)^14^. Type B strains carry plasmids which encode one of the deadliest toxins on earth, ε-toxin, and β-toxin. Plasmid pCP8533etx encodes *etx* and *cpb2* genes, but not *cpb*^18^. Most plasmids in type B strains also possess other virulence genes encoding λ-toxin and urease^19^. Similarly, plasmids in type D strains also carry *cpe* and/or *cpb2* genes, and plasmids range in size from 45 to 110 kb^20^. *C. perfringens* type C strains possess plasmid-borne β-toxin gene *cpb*, and other plasmids reported to carry other toxin genes including *cpb2*, *tpeL*, or *cpe*^21^. In type E strains, two major families of plasmids have been identified: (1) plasmids carry *iap/ibp* genes, λ-toxin gene and *cpe* gene, however, *cpe* contains nonsense and missense mutations in the *cpe* ORF and is thus non-functional^22^. (2) These plasmids, including pCPPB-1, encode both *iap/ibp* genes and *cpe* gene but not the λ-toxin gene^23^. NetB is a chicken NE-associated toxin, which is plasmid encoded. These *netB*-encoded plasmids can potentially carry other virulence genes including tetracycline resistance genes (e.g., pJIR3537 and pCW3) and *cpb2* gene (e.g., pJIR3844)^24^.

Notably, many toxin plasmids of *C. perfringens* are highly similar, sharing up to 35–40 kb of identical sequences^15^. In addition, some *C. perfringens* isolates can carry multiple near-identical plasmids that encode different toxin and AMR genes^24^. However, no one isolate has ever been found to possess plasmids that encode all key toxins; only certain plasmid combination could can be maintained, which suggests plasmid incompatibilities, due to presence of distinct plasmid segregation systems, and warrants further research^14,25,26^. Furthermore, the universal existence of *tcp* locus indicates that conjugative plasmid transfer could be a key HGT event for increasing virulence of *C. perfringens* strains.

## Virulence-related factors

**Fig. 3:**
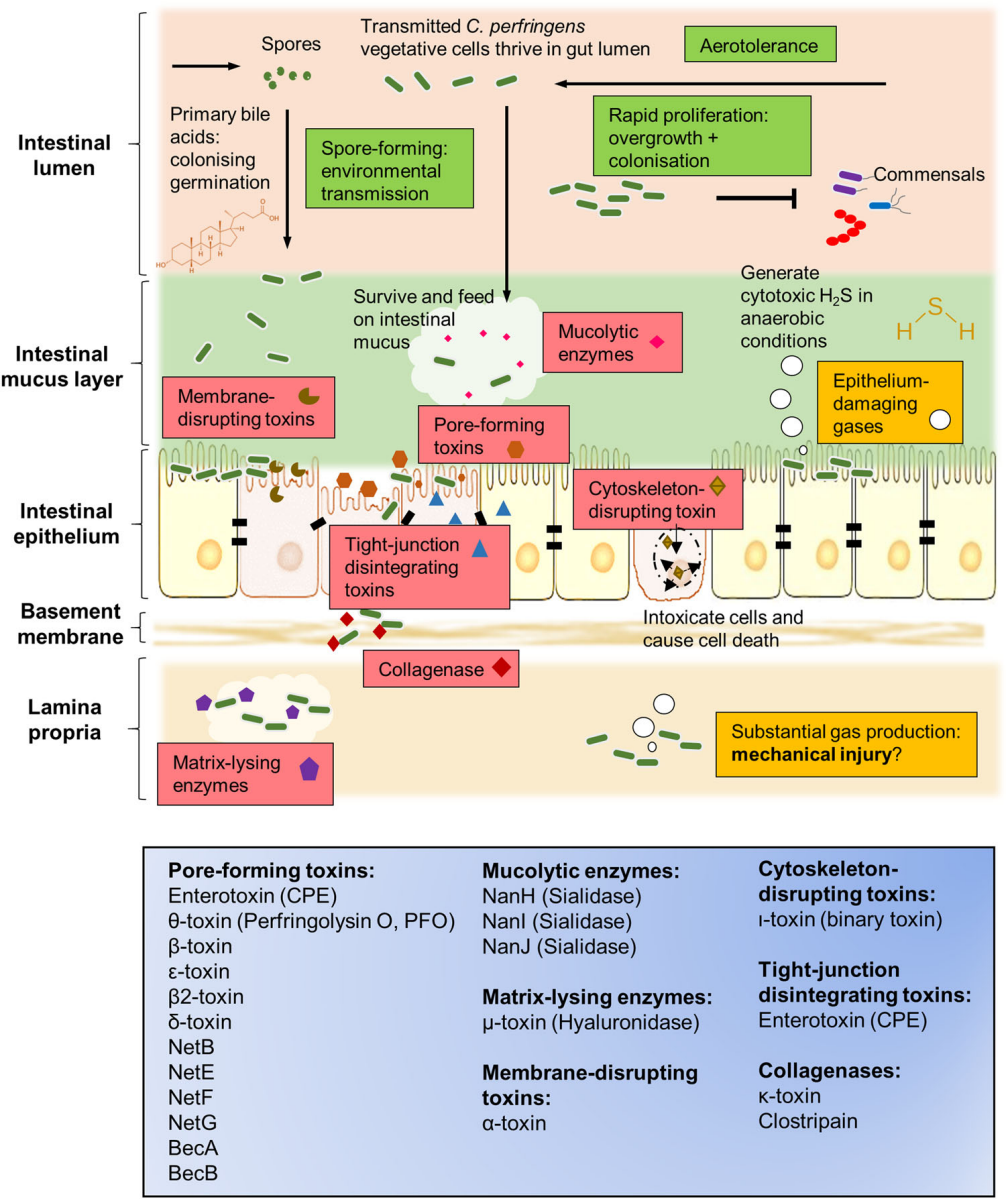
Graphical representation of disease-linked virulence factors of *C. perfringens* in the context of intestinal infections. Classification of toxins according to general mechanisms of action are shown in the bottom blue box

*C. perfringens* can generate a complement of extracellular toxins and hydrolytic enzymes (>20), survive in aerobic environments (i.e., oxygen tolerance), produce toxic gases, and rapidly grow. Therefore, it possesses the capacity to effect various histotoxic (i.e., toxigenic to tissues) infections in humans, including gas gangrene in contaminated wounds, gastroenteritis (including foodborne and non-foodborne diarrhoea) in human adults, necrotic enteritis (NE) in animals, and recent links to NEC in pre-term infants^27^. Relevant virulence factors of *C. perfringens* are represented and summarised in Fig. 3.

## Transmission and colonisation

### Oxygen sensitivity

*C. perfringens* is commonly known as a strict anaerobe; however, this bacterium can also survive in the presence of oxygen, and/or low concentrations of superoxide or hydroxyl-radical-generating compounds^28^. Notably, as an aero-tolerant anaerobe, *C. perfringens* could potentially survive through aerobic environments (such as on surfaces in hospital wards^29^) and trigger disease development in aerophillic environments (i.e., adult/pre-term infant gut), and additionally oxygen-exposed tissues (such as gas gangrene), which may facilitate bacterial host-to-host transmission^30^.

### Sporulation

*C. perfringens* is a spore-former, which enables this bacterium to survive in extreme or nutrient-depleted conditions. This spore-forming characteristic plays a vital role in the transmission of this Gram-positive bacterium from diverse environments to hosts, leading to infection, including food poisoning in human adults^31^. Some spores of *C. perfringens* (especially food-poisoning associated strains) had been demonstrated to resist extreme temperature conditions, which may contribute to *C. perfringens* survival and subsequent disease pathology^32,33^. Molecular regulation of sporulation in *C. perfringens* is less well studied compared to the model bacterium *Bacillus subtilis*. Previous studies have indicated that the transcriptional regulator CcpA (encoded by *ccpA*) is required for effectual sporulation^34^ and Spo0A (*spo0A*), a transcriptional factor known to be present in *Clostridium* species including *C. perfringens*, is associated with sporulation initiation^35^. Sigma factors, including SigF, SigE, SigG and SigK (genes *sigF*, *sigE*, *sigG* and *sigK* respectively) are known to regulate the sporulation process of *C. perfringens*^36,37^. In addition to this, it was also reported that *C. perfringens* sporulation processes could be regulated by an Agr-like quorum-sensing system, potentially promoted by the *codY* gene in type A food-poisoning strains^38,39^. Importantly, CPE is known to be produced by *C. perfringens* during sporulation, which correlates with the pathogenesis of food poisoning-associated diarrhoea^31^.

### Germination

Germination of *C. perfringens* can be triggered by small molecules termed germinants—commonly known as sugars, nucleosides, amino acids, salts and purines^40^. Importantly, primary bile acids (including glycocholate, cholate and taurocholate) in the human GI tract are also known to act as potent germinants in *Clostridium* species, whereas secondary bile acids, such as deoxycholate (derivatives generated by certain gut microbes), can inhibit the in vitro proliferation of *C. perfringens*^41^. This proposed ‘colonising germination’ survival modus operandi of spore-formers, including *C. perfringens*, potentially acts as a signal to initiate growth, and to persist in the intestinal environment^30^. This persistence, and long-term gut colonisation may correlate with the ongoing symptoms (up to several weeks) of nonfoodborne diarrhoea in patients^42^. From a colonisation resistance perspective, inhibition of germination, by secondary bile products produced by resident commensal bacteria, implies a long-standing competition for limited nutrients and niches within the gut.

### Rapid proliferation

Notably, *C. perfringens* represents one of the fastest-growing organisms currently known, and is reported to have a very short 8–12 min generation time when cultured at 43 °C in optimal media, and 12–17 min at 37 °C^43^. Rapid growth of this bacterium potentially predisposes the host to tissue infection, as in the cases of avian NE, bovine necro-haemorrhagic enteritis (could be <5 h), and gas gangrene. Also, as *C. perfringens* has a two-fold quicker generation time, when compared to intestinal commensals like wild-type *Escherichia coli* (typically 20–30 min in Luria-Bertani broth), this could represent a potent mechanism for outgrowing other resident bacteria, leading to efficient gut colonisation.

## Disease initiations

### Histotoxic gas production

Clostridial myonecrosis (better known as gas gangrene), is accompanied by profuse gas production, which could in theory be the mechanical determinant of tissue injury, alongside rapid cell proliferation of *C. perfringens*. Importantly, *C. perfringens* is known to generate hydrogen sulphide^44^ (using ubiquitous sulphuric sources from the environment), a readily-permeable toxic substance to human cells, which when produced in excess is associated with other intestinal inflammatory diseases (e.g., Ulcerative Colitis)^45^. Thus, *C. perfringens*-associated gas production represents an understudied virulence trait in the pathogenesis of gut infection, such as infant NEC (discussed in more detail below), which symptoms significantly mimic those of gas gangrene; necrosis of tissue accompanied by abundant gas production^46,47^.

### Toxins and virulent enzymes

Presently, 23 virulence genes that encode toxins and virulent enzymes have been identified in *C. perfringens* (summarised in Table S1); the most ‘prolific’ toxin-producing pathogen currently known. Some key toxins will be briefly discussed and summarised in this sub-section.

#### Alpha-toxin

Alpha-toxin (encoded by gene *plc* or *cpa*), which is produced by all strains of *C. perfringens*, has been shown to hydrolyse cell membrane phospholipids, which eventually leads to cell necrosis; a key characteristic in gas gangrene. Indeed, this toxin is essential for gas gangrene pathology, as shown in an in vivo myonecrosis model^48^. Mechanistically, α-toxin may play three major roles in gas gangrene pathology; firstly, it is able to impact the transferring of immune cells such as neutrophils to infected tissues (mechanism currently unknown), hence potentially reducing pathogen clearance at infected sites. Secondly, it can cause constriction of blood vessels, which may reduce the blood supply to tissues, thus creating a micro-aerophillic environment conducive to *C. perfringens* overgrowth. Thirdly, this toxin can activate inflammation cascades in host cell metabolism (arachidonic acid and protein kinase C), which may lead to direct immune-mediated pathology of tissues^49^.

#### Beta-toxin

Beta-toxin, encoded by gene *cpb*, is a plasmid-encoded pore-forming toxin that is thought to be intestinal-necrotic, and also important for systemic enterotoxaemia, in humans and neonatal animals, as shown in in vivo studies^50,51^. *C. perfringens* type C strains that possess the *cpb* gene were associated with historical Clostridial gut infections; Darmbrand (post-world war II in Germany), and Pig Bel (Papua New Guinea)^52^. Previous intestinal loop studies have indicated a synergistic effect with CPE (encoded by *cpe*)^53^. Furthermore, this toxin shares sequence similarity with several pore-forming toxins produced by *Staphylococcus aureus* – ɣ toxin (A: 22 and B:28%), α-toxin (28%) and leucocidin (S:17% and F:28%)^54^.

#### Perfringolysin O

PFO (also θ-toxin, encoded by gene *pfoA* or *pfo*), is also a pore-forming toxin that acts on cholesterol-comprising cell membranes. This toxin has been shown to be involved in the pathogenesis of gas gangrene, and haemorrhagic enteritis in calves^55,56^. Notably, PFO shares structural homology with similar pore-forming toxins identified in *Streptococcus*, *Bacillus*, *Listeria* and many other genera^57^. θ-toxin is also known for its capacity to induce tumour necrosis factor alpha (TNF-α) and interleukin 6 (IL-6) expression in the host, and it could activate apoptosis through p38 MAPK (mitogen-activated protein kinase) pathway as demonstrated in in vitro models^58^. Interestingly, it has synergistic cytotoxic effects with α-toxin on bovine epithelial cells, highlighting the significant role of this toxin in disease development^56,59^.

#### Beta2-toxin

Beta2-toxin (encoded by *cpb2* plasmid gene), a pore-forming cytolytic toxin shares <15% sequence homology with β-toxin, distinguished itself as a novel toxin produced by *C. perfringens*^54^. β2-toxin-producing *C. perfringens* strains have been associated with gut diseases such as NE in piglets, and enterocolitis in foals^60^. Importantly, this pore-forming toxin has been suggested as an accessory toxin in *C. perfringens*-associated non-foodborne diarrhoea^61^, and proposed to play an important role in pre-term NEC in potential synergistic effects with antibiotic gentamicin^62^.

#### Epsilon-toxin

This deadly toxin, is plasmid-encoded by *etx* gene *C. perfringens* type B and D strains, and is essential for pathogenesis. Epsilon toxin is involved in animal (goat and sheep) enterotoxaemia^63^. ε-toxin is currently thought to be the most potent of all toxins produced by *C. perfringens*, evidenced by the LD_50_ of 70 ng/kg body weight, ranked only behind *C. botulinum* and *C. tetani* neurotoxins. It has been demonstrated to affect various organs, such as kidneys and liver, through unknown mechanisms, that allow this toxin to enter systemic circulation^64^. Due to its potential use as a biological weapon, ε-toxin-producing *C. perfringens* strains are on the export control list in a number of countries including USA and the UK.

#### Iota-toxin

This cytoskeleton-damaging toxin (also considered as a major toxin produced by *C. perfringens*), consists of two cell-binding monomers Ia (enzymatic component) and Ib (binding component; encoded on plasmids by *iap* and *ibp* gene respectively), which act synergistically to first translocate the toxin into host cells, then exert enzymatic activity on ADP-ribosylating actin to disassemble the cytoskeleton, which eventually leads to apoptosis and cell death^65^.

#### Microbial collagenase

Microbial collagenase (also known as κ-toxin), encoded by chromosomal gene *colA*, is a key toxin produced by *C. perfringens* that degrades collagen. This enzyme might contribute to intestinal infection, as collagen, the substrate for collagenase, is a primary component of intestinal connective tissues/basal membrane of human and animal hosts, thus disruption may damage basal integrity, which may lead to eventual tissue necrosis^56,66^. However, Awad et al.^67^ indicated that κ-toxin is not a major determinant in a Clostridial myonecrosis mouse model (gas gangrene), despite its capacity to hydrolyse collagen.

#### Enterotoxin (CPE)

CPE (encoding gene *cpe*) is recognised as the key toxin to cause food-poisoning and non-foodborne diarrhoea, it has also been demonstrated to disrupt the intercellular claudin tight junctions in gut epithelial cells^68,69^. Importantly, this pore-forming toxin was demonstrated to bind and necrotise human ileal and colonic epithelium in vitro, and can induce cell death i.e., apoptosis, via the caspase-3 pathway^70^. Hence, the potential pathogenesis mechanisms underlying food poisoning could result from CPE-induced tight junction rearrangements or pore-formation.

#### Sialidase

Three sialidases, encoded by genes *nanH, nanI* and *nanJ* in *C. perfringens*, are also named as neuraminidases or exo-sialidases (NanI and NanJ). This group of enzymes represent important virulence factors during *C. perfringens*-mediated tissue infection; they catalyse hydrolysis of terminal sialic acids from glycoprotein, glycolipids and polysaccharides of cell membranes that aids in bacterial attachment to host cells^71^. This mucolytic potential suggests that *C. perfringens* may utilise intestinal mucus as a nutrient source, and thereby potentiates intestinal colonisation, which has been modelled using the in vitro Caco-2 cell line^72^. Importantly, in vitro studies have also demonstrated that α-toxin associated with NanI (exo-alpha-sialidase) increased the virulence of *C. perfringens*^73^. Furthermore, NanI was shown to potentiate the virulence of ε-toxin, β-toxin and CPE, via binding-enhancing (ε-toxin) and proteolytic activation (β-toxin and CPE) mechanisms, potentially enhancing *C. perfringens* pathogenesis^74^. Notably, in a gas gangrene mouse model, NanI and NanJ are not essential for virulence^75^.

#### NetB

NetB, a pore-forming toxin (encoded by *netB*), shares limited amino acid sequence similarity with beta-toxin (38% sequence identity), α-toxin from *S. aureus* (31% sequence identity) and δ-toxin (40% sequence identity) from *C. perfringens*^76^. Less than a decade ago, this chicken-NE associated toxin (plasmid-borne), was discovered and shown to be essential (instead of α-toxin) for lesion formation both in cell line models, and avian in vivo models via genetic mutant strains, and thus fulfilled molecular Koch’s postulates as a disease determinant^77^. This toxin is important in avian agriculture as *netB*-positive *C. perfringens* strains recovered from broiler chickens (healthy birds) can be as high as 61%, however expression of NetB was shown to be lower in healthy birds, when compared with NE-associated chickens (92% vs. 29%)^78^. In addition, *netB* was later identified as part of a plasmid-encoded pathogenicity locus named NELoc-1 capable of being transferred via conjugation, indicating the potential spread in relation to chicken NE epidemiology^24,79^. Please refer to Review by Rood et al.^80^ for a comprehensive description on NetB and poultry NE.

These extracellular toxins represent potent virulence factors central to intestinal disease development. However, not all toxins are secreted by all strains of *C. perfringens* (excluding α-toxin), hence, in silico identification of virulence genes using NGS techniques and bioinformatics tools are essential for rapid and comprehensive geno-toxinotyping of *C. perfringens*, compared with conventional molecular tools.

## Antimicrobial resistance (AMR)

AMR traits in *C. perfringens* pose a serious clinical treatment concern, due to its capacity to generate an array of lethal toxins. Presently there are several phenotyping studies published (based on in vitro susceptibility testing of minimal inhibitory concentration; MIC, Table S2) on the AMR profile of *C. perfringens*, however, there is currently limited genetic/WGS-based AMR genotyping studies.

Tetracycline resistance in *C. perfringens* was first described in 1968, when 11 strains of *C. perfringens* were tested, and found to possess ‘some degree’ of resistance to tetracycline, thus penicillin G (or, benzylpenicillin) was recommended as the drug of choice for treating Clostridial infection^81^. Plasmid-carrying tetracycline resistance (*tet* components) in *C. perfringens* was genetically shown for the first time in porcine samples (three strains)^82^. Tetracycline resistance elements *tetA*(P), was then detected in 81 tetracycline-resistance *C. perfringens* strains (100%), with 93% of these strains carrying a secondary resistance gene, either *tet*B(P) or *tet*(M), notably, no single strain possesses all *tet* genes^83^. Multidrug-resistant *C. perfringens* strains were first reported back in 1977, multiple strains (*n* = 7) isolated from porcine faeces were demonstrated to be resistant against tetracycline (MIC > 32 μg/ml), erythromycin (MIC > 128 μg/ml), clindamycin (MIC > 64 μg/ml), and lincomycin (MIC > 128 μg/ml)^84^. A PCR-based AMR study on 160 environmental strains (water, soil and sewage) revealed encoded macrolide resistance genes *erm*(B)(26%), *erm*(Q)(1%), and *mef*(A)(18%), in addition to the commonly found *tetA*(P)(53%), *tetB*(P)(22%), and *tet*(M)(8%)^85^.

Macrolide and Lincosamide resistance (mainly erythromycin and lincomycin) appears widespread^86^, and therefore is considered ineffective in treating *C. perfringens* infections. This is supported by a recent multidrug-resistance study of 260 strains of *C. perfringens* isolated from diarrheal neonatal piglets in Thailand, where higher resistance was observed for erythromycin, lincomycin and tylosin^87^.

Recent WGS studies on AMR genes have detected *mepA* (multidrug-resistance gene), using various public AMR databases on *C. perfringens* strains^88^, and also tetracycline resistant genes *tetA*(P), *tetB*(P) and *tet38*. The genes, *mprF* and *rpoB* (rifampin-resistant) have also been reported to be encoded^88^. Anti-defensin gene *mprF* (possibly involved in multidrug-resistant, including resistance against gentamicin) was recently reported in a large-scale genomic study of *C. perfringens* (*n* = 56 strains) to be present in 100% of the genomes^10^. In this latest genomic study, *tetA*(P) was detected in 75% of the 56 strains, which is more prevalent than *tetB*(P)(42%). Interestingly, *ANT(6)-Ib*, an aminoglycoside resistance gene, was also reported to be encoded in a *C. perfringens* toxinotype C strain. Although mainly anaerobic bacteria like *C. perfringens* may have reduced transport of aminoglycosides intracellularly, there are also findings that *C. perfringens* are sensitive to aminoglycosides like Gentamicin at higher concentration, which indicates that *C. perfringens* might also have acquired additional resistance to aminoglycosides^89,90^.

WGS will be a key tool in the fight against *C. perfringens* AMR, particularly in rapid diagnostics, however gold-standard phenotypic MIC tests will also be required to clinically determine the antimicrobial susceptibilities, and facilitate antibiotic management.

## Clinical associations in humans and animals

*C. perfringens* has been constantly associated with gut diseases across both animal and human hosts (summarised in Table S3) as described in this section.

## Animal hosts

### Poultry NE

Poultry NE was first documented in England in 1961^91^. Since then, NE has been consistently reported in every continent around the globe. Importantly, *C. perfringens* is unequivocally identified as the key aetiological organism of NE in broiler chickens^92^. Global financial impact of NE is significant, with an estimated economic loss of 6 billion US$ in 2015, projecting profit loss per bird at >US$0.062^93^.

Classic NE pathologies are characterised by gaseous lesions and mucosa necrosis in gas-filled distended small intestine, however, these can also involve kidney and liver as secondary infection sites^94^. Proposed key biological factors that contribute to NE are hydrolytic enzymes (e.g., collagenase)^95^, toxin production (traditionally understood as α-toxin, and more recently NetB and TpeL, both are pore-forming toxins, were linked to the onset of NE^96^. Other NE-predisposing factors include: a high-protein diet that favours the growth of *C. perfringens*, and environmental stress (e.g., high stocking density), which alters gut microbiota/host immunity that eventually increases risk of infection^97^.

### Equine acute necrotising enterocolitis

Acute necrotising enterocolitis (ANEC) in foals/horses (caused by *C. perfringens*) is a severe intestinal disease that resembles the classic clinical signs of *C. perfringens*-infections; rapid disease development and necrotic intestines (mainly the colon)^98^. ANEC symptoms are characterised by bloody diarrhoea, followed by a haemorrhagic and necrotic gut^99^. Type F *C. perfringens* strains (previously type A) harbouring both CPE and β2-toxin are often associated with this deadly condition^98^. More recently, NetE, NetF and NetG toxins (pore-forming toxins) were proposed to underlie the pathogenesis of foal NEC^100^.

### Swine enterocolitis

*C. perfringens* type A strains (also less frequently, CPE-harbouring type F strains) are widely considered as the invasive agent of non-haemorrhagic EC in piglets, although the actual pathogenesis remains undefined. Similar to other *C. perfringens*-intestinal infection, this disease commonly affects one-week-old piglets, suspected to inherit (i.e., via vertical transmission during birth) the bacterium from the microbiota of mother sows^101^. Symptoms involve severe diarrhoea (non-haemorrhagic), accompanied by necrotic mucosa and atrophy of intestinal villi. β2-toxin was initially believed to drive the development of this disease (backed by epidemiological studies in 1990s), and as such was used as a diagnostic biomarker, although this has become controversial in recent years^60^. Type C strains that carry β-toxin are associated with haemorrhagic EC in piglets and largely affect 1 to 4 days old neonates^101^. In contrast to type A-infection (to a lesser degree, type F-infection), type C-infection is characterised by haemorrhagic NE, which is proposed to be driven by the presence of type C strains and low trypsin secretion (trypsin can inactivate β-toxin) in the immature host gut^102^.

## Human hosts

### Darmbrand

Darmbrand, which literally means ‘burning (fire) bowels’ in German, was used to describe a particular type of necrotic inflammatory gut disease (also known as enteritis necroticans, EN) associated with *C. perfringens*, that occurred epidemically post-second World War (1944–1949) in north-west Germany^103^. Darmbrand causative strains were later classified as type C strains that carry β-toxin, and these strains are highly resistant to heat^104^. Many of these type C strains also harbour CPE, which has been shown to act synergistically with β-toxin in an intestinal loop model and therefore CPE was suggested to play a role in some cases of EN^53^. Notably, the heat-resistance of this strain might be attributed to the production of a small acid-soluble protein (Ssp4) that could potentially play a central role in dissemination of *C. perfringens* strains^33^. Darmbrand was believed to be facilitated by poor post-war sanitary (bacterial contamination), and malnutrition (protein-malnourished) conditions, as this disease disappeared within a few years after its first recognition.

### Pigbel

Enteritis necroticans (EN), or commonly known as pig-bel in the highlands of Papua New Guinea (PNG), is a form of inflammatory gut disease that was associated with pork-feasting activities that took place among PNG Highlanders in outbreaks first documented back in 1966^105^. The classical description of the symptoms is ‘spontaneous gangrene of small intestine, without obvious vascular or mechanical cause’ which resembles Darmbrand, and occurred particularly in children^106^. The aetiology of this fatal infection was thought to be large co-consumption of *C. perfringens* type C-contaminated pork (due to poor hygiene), and large amount of sweet potatoes which contain trypsin inhibitor (trypsin secreted in the gut could break down β-toxin secreted by *C. perfringens*)^107^. Similar cases of EN were also reported in Uganda^108^ and Indonesia^109^. Notably, EN-like cases were also reported (rare cases) in developed countries, including USA, in exclusively diabetic subjects, who also have attenuated trypsin production^110^.

### Acute watery diarrhoea (food poisoning)

*C. perfringens* has been associated with food poisoning since it was first documented in both the UK and the USA in the 1940s^111^. *C. perfringens*-type A food poisoning, is ranked the second most prevalent bacterial food poisoning in the US, estimated at 1 million cases per annum, after *Salmonella*^112^. In the European Union (EU) member countries, *C. perfringens*-linked food-poisoning outbreaks were projected at approximately 5 million cases per year (2011)^113^. *C. perfringens* has also been reported to affect elderly communities, especially in care homes (North East of England, 2012–2014; 83% of the outbreaks reported from care homes)^114^. *C. perfringens*-linked foodborne cases are suggested to be under-reported due to its self-limiting symptoms, thus the statistics published based on laboratory-confirmed cases may be lower than the actual numbers, suggesting a higher actual epidemiological impact.

Hallmark symptoms (self-limiting, lasting 12–24 h; mortality is uncommon) include, rapid appearance within 8–14 h after food ingestion, intestinal cramp, watery diarrhoea without fever or vomiting^115^. Currently, food poisoning *C. perfringens* cases are thought to be caused by CPE (encoded by *cpe* gene). This secreted pore-forming toxin is also demonstrated to disrupt intestinal tight junction barriers and initiate disease development^68^.

### Non-foodborne diarrhoea

*C. perfringens* has also been associated with non-foodborne diarrhoea (a distinct disease entity from food poisoning, characterised primarily by more severe symptoms and longer duration), which includes antibiotic-associated diarrhoea (AAD), and sporadic diarrhoea (SD)^116,117^. AAD typically occurs in 5–25% of patients after administration of broad-spectrum antibiotics^118^. Non-foodborne diarrhoea typically affects older adults that are >60 years of age (although SD is also associated with younger age groups)^119^. Clinical symptoms are abdominal pain, prolonged diarrhoea (>3 days to several weeks), and bloody stools^120,121^. Patients suffering from non-foodborne diarrhoea, particularly AAD, can become seriously ill due to diarrhoea-induced dehydration, and may progress to develop colitis, although full recovery is common^122^.

Although other pathogenic bacteria including *C. difficile* (most common AAD pathogen; ~25% of cases^123^) and *S. aureus* have also been implicated in AAD, enterotoxigenic *C. perfringens* type F strains (producing CPE) are estimated to be responsible for up to 15% of all AAD cases^116,124,125^. CPE, produced by *C. perfringens* type F strains (encoded by plasmid-borne *cpe* gene), has been reported to be the aetiological agent for *C. perfringens*-associated non-foodborne diarrhoea, as evidenced by the high prevalence in diarrhoea patients, but not healthy individuals. Importantly, type F strains have also been linked to SD, although to a lesser extent^116^. AAD-associated *C. perfringens* strains are also described to be more adherent to Caco-2 intestinal cells, when compared to other food-poisoning strains, which is attributed to the production of sialidase NanI^126^. The spore-forming nature of *C. perfringens* could also potentially contribute to the commonly observed disease persistence and relapse^127^. Molecular detection of faecal CPE or PCR confirmation of *cpe* gene represents the current clinical diagnostic method for *C. perfringens*-AAD^128,129^.

### Pre-term infant NEC

Pre-term infant NEC has been clinically linked with *C. perfringens* since the 1970s, as type A-*C. perfringens* were isolated from necrotic tissues in many NEC cases. Notably, it was even called ‘gas gangrene of the bowel’ owing to its reflection of the highly similar diseased histology with the infamous tissue myonecrosis^47^.

NEC has ~14% prevalence in pre-term infants less than 1000 g birth weight (i.e., extremely low birth weight, ELBW), a high mortality of 30% with surgical NEC, and mortality up to 50%. NEC-related deaths account for 10% of all mortality causes in neonatal intensive care units (NICUs) according to a UK-nationwide study^130^, and is the most severe and lethal neonatal GI emergency worldwide^131^. The additional annual cost for treating NEC in pre-term infants is conservatively estimated at £13.5 million in the UK (excluding long-term post-surgical treatment), whilst in the US $1 billion is estimated to be spent annually by neonatal departments on NEC^132^.

The involvement of bacteria in the pathogenesis of NEC has been strongly implicated since the disease was first described^133^. Both *Klebsiella* spp., and *C. perfringens*, have been linked to clinical NEC in recent years^4^. Importantly, *C. perfringens* was isolated from most neonates (70%), as early as the fifth day of life, which supports the theory of pathogen colonisation that then leads to microbial invasion. Classic NEC symptoms such as pneumatosis intestinalis, suggests gas-producing Gram-positive bacteria including *Clostridium* spp. (also implicated consistently in recent metagenomics studies), as the key causative agent^134,135^. Most recently, the emergence of two extensive pre-term infant NEC cohort studies that profiled metagenomic faecal samples, have both indicated that *C. perfringens* is significantly abundant in the infant gut microbiota prior to NEC development^4,5^. These studies are supported by the substantial number of NEC studies (14 studies to date since 1970s, more than any individual bacterial agents, as summarised in Table S4) that have reported *C. perfringens* as a potential pathogenic agent, in addition to its established pathogenesis in many other neonatal animal gut diseases, and further supported by in vivo studies^136^. Thus, *C. perfringens* appears to be a pathogenic agent of microbial-NEC, i.e., *C. perfringens*-associated NEC, and may link to specific disease symptoms^137^.

**Fig. 4:**
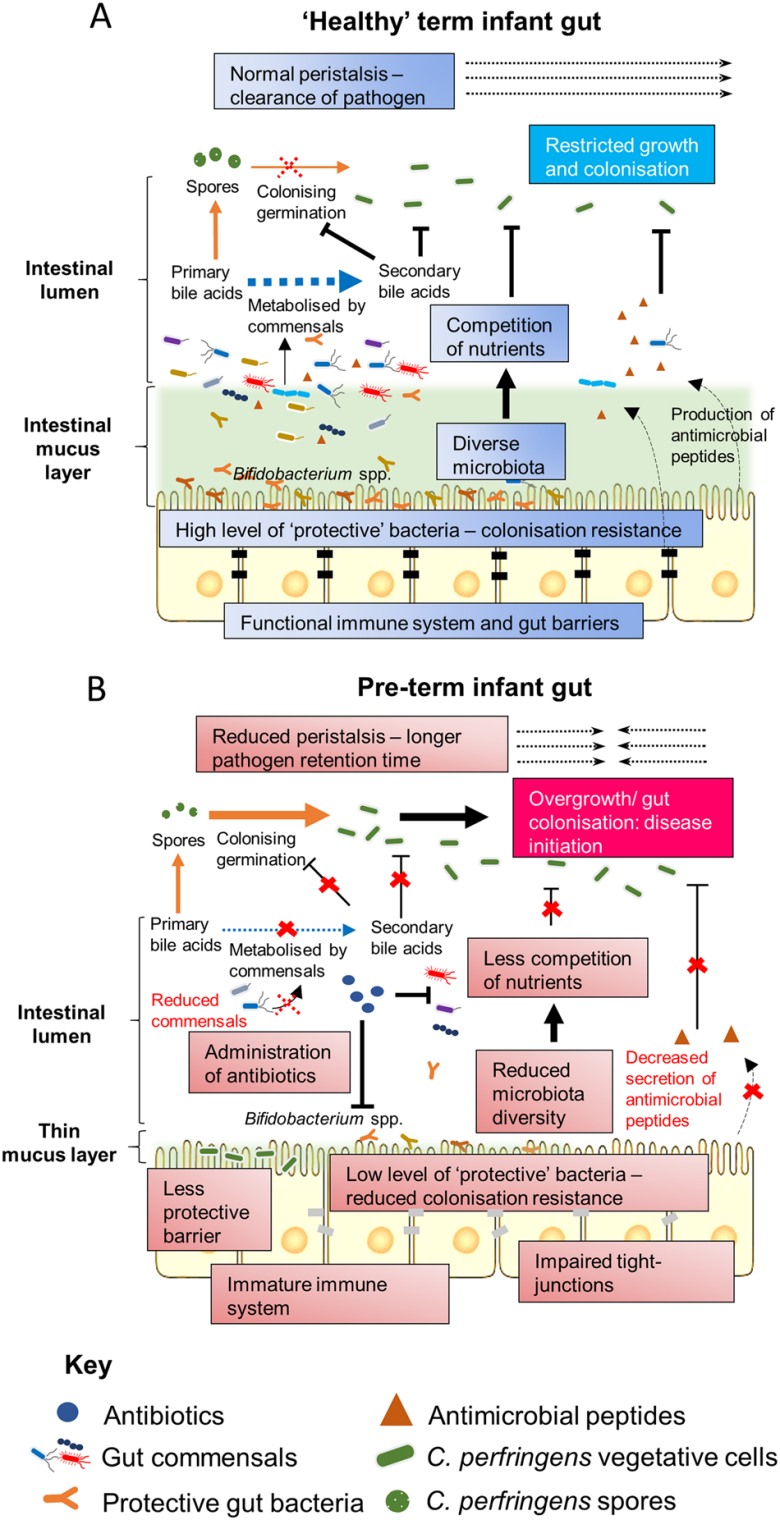
Proposed infection mechanisms that underlie *C. perfringens*-associated NEC. **a** In non-NEC ‘healthy’ term infant gut. **b** In pre-term infant gut that leads to *C. perfringens*-associated NEC

Being universal and resilient in habitable niches, spore former *C. perfringens* may readily pass on to in-hospital neonates through either environmental or oral transmission, and then initiate diseases in the intestine^138^. Proposed underlying mechanisms for *C. perfringens*-associated NEC include reduced bowel peristalsis in pre-term infants (increased pathogen retention time), universal administration of broad-spectrum antibiotics^139^ (that leads to reduced microbiota diversity, and the potential rapid overgrowth of resistant spores), reduced gut barrier integrity (non-existent mucus layer), lack of protective bacteria^4,5,131^ (including *Bifidobacterium* species, which may antagonise the growth of *C. perfringens*), and immature/underdeveloped immune system that is ineffective at fighting off pathogenic bacteria^46^ (Fig. 4). Bile salt factors may also be linked to disease onset. Reduced diversity of the microbiota in pre-term infants, including bacteria that can de-conjugate bile salts to secondary bile acids, may allow germination of *C. perfringens*, which has also been directly indicated in the pathogenesis of the well-studied *C. difficile* colitis (both species form spores and secrete colitis-related toxins)^140^.

To date, no particular toxin(s) has been specifically linked with pre-term NEC, although β2-toxin has been suggested^4^. Notably, *C. perfringens* can also be part of a ‘healthy’ human microbiota (as low as 3.5% in pre-term neonates, nevertheless this is controversial^141^), thus suggesting other factors play a role. Antibiotic administration in pre-term babies is very high, with up to 97% of pre-term infants residing in NICU exposed to at least one dosage of broad-spectrum antibiotics^142^. These antibiotics eliminate ‘protective’ members of the gut microbiota, and in tandem with presence of AMR-*C. perfringens*, may facilitate successful colonisation and infection^143^. An immature gut barrier in neonatal infants may also expose gut niches for overgrowth, and finally the presence of ‘hyper-virulent’ strains of *C. perfringens* may be implicated. However, to comprehensibly answer these important questions a more global genomic approach, i.e., WGS and analysis, and validation using suitable in vivo models is required.

Advancement of WGS and genomic analyses, particularly those focussing on other gut pathogens including *C. difficile*, *K. pneumoniae* and *Salmonella enterica* has proved successful in understanding the pathogenic role of these bacteria in disease development and tracking bacterial transmission^144,145^. These enteric pathogens have all been sequenced extensively with genomic information made public on NCBI database (as of May 2018; bracketed digits indicate number of genomes): *C. difficile* (1322), *E. coli* (10541), *C. botulinum* (225), *K. pneumoniae* (3927) and *S. enterica* (8780). Yet, including a batch addition of 30 genomes towards the end of 2016, there are only 59 publicly available genomes of *C. perfringens* (May 2018), highlighting the lack of in-depth understanding of this pathogen with respect to its genomic information. Importantly, no NEC-associated pre-term infant isolates are known to be sequenced at present, and genomic investigation may allow identification of putative/novel NEC-associated virulence factors, that may facilitate intervention strategies and/or therapy development.

## Future research directions

The clinical link between intestinal diseases and *C. perfringens* is clear and defined, however the underlying factors responsible for specific aspects of pathology remains uncertain (Box 1, Box 2). Deciphering the genes that are involved in the sporulation, germination, enzyme/toxin production, oxygen tolerance, AMR and other novel virulence factors could lead to more targeted clinical preventions in *C. perfringens*-associated adult and neonatal intestinal diseases, whether it be humans or animals. Important questions may be answered utilising WGS and in silico tools, to delve into the genetic makeup of this notorious, yet under-studied pathogen. In tandem further in vitro and in vivo studies should be carried out to confirm the importance of suspected novel virulence traits, i.e., to fulfil Molecular Koch’s postulates. These approaches may help establish future platforms for disease prevention strategies such as vaccines, phage therapy, microbiota-based therapeutics, or implementation of specific antibiotic administration policies. Furthermore, understanding the genomes will potentially enable epidemiological tracking (as is taking place for other pathogenic bacteria), allowing us to pinpoint the origin and route of spread of isolates in the hospital settings, which is vital within clinical contexts.

Box 1 Glossary

- 16S rRNA gene: a housekeeping gene (of ribosomes) that is conserved across all species therefore is widely used as a chronological/biological identity marker in evolutionary genetics and genomics.
- Accessory gene: a gene that is absent in one or more strains in an analysed pangenome.
- Antimicrobial resistance: a biological characteristic that enable a microorganism to survive and thrive in the presence of antibiotics.
- Core gene: a gene that is present in all strains in an analysed pangenome.
- Genotyping: a process to differentiate/identify characteristics of genes (genotypes).
- Germination: the process which microorganisms grow from a dormant state (usually from endospores).
- Necrotising Enterocolitis: severe gut inflammation (often accompanied by necrosis of the gut) which occurs mostly in pre-term infants with high mortality.
- Pangenome: The repertoire of collective genes in closely related organisms.
- Phenotyping: a process to observe the expression/traits (phenotypes) of an organism’s genotype.
- Sporulation: a process certain species/genus of bacteria (e.g., Firmicutes) undergo to produce a tough external structure (spore/endospore) to withstand unfavourable conditions in order to survive through.

Box 2 What are the key knowledge gaps in understanding *C. perfringens* as a pathogen, and what are the potential approaches to investigate/mitigate the virulence of *C. perfringens* in clinical diseases?

Questions

- Compared to other pathogens (e.g., *C. difficile*), there is limited WGS data for *C. perfringens*, what is the genomic diversity of this important pathogen?
- Can we use WGS to ‘type’ *C. perfringens* isolates, and can WGS be used in diagnostics (including AMR profiling), and to track spread in outbreaks settings?
- *C. perfringens* causes diverse diseases in a wide host range, but what are the microbial virulence factors responsible for pathology, and is this host/strain specific?
- What are the host immune cells/signalling cascades involved in *C. perfringens*-associated clearance and/or pathology?
- *C. perfringens* can reside as a ‘normal’ member of the gut microbiota, what are the external factors that may lead to overgrowth and disease?
- Can *C. perfringens* be directly linked to NEC pathogenesis?
- What are the therapeutic approaches that could be used to prevent *C. perfringens* infection in multi-host species?
  Approaches
- Isolate diverse strains from various environments, i.e., from both case and control samples
- Perform comprehensive bioinformatics analyses to understand evolution, e.g., SNP based phylogeny, and investigate variants and spread clinical settings
- Carry out pan-GWAS/comparative genomics to identify/predict specific virulence genes related to disease development.
- Develop/optimise clinical diagnostics from faecal samples via e.g. ultra-long read real-time sequencing method such as Oxford Nanopore
- Develop and characterise in vivo infection models for different disease types (e.g., NEC and gastroenteritis)
- Pinpoint specific virulence genes involved in infection via bacterial transcriptomics (RNAseq) and knock-out bacterial strains, to fulfil molecular Koch’s postulates.
- Perform microbiota profiling (e.g., 16S rRNA metagenomics or shotgun metagenomics) to investigate the impact of *C. perfringens* to microbiota members, and the role of microbiome in *C. perfringens* infections
- Understand host defence mechanisms using immunological approaches, and knock out in vivo models
- Develop therapeutics for *C. perfringens* infections including phage therapy, vaccines, microbiota treatment i.e., probiotics.

## Electronic supplementary material

Supplementary Table S1

Supplementary Table S2

Supplementary Table S3

Supplementary Table S4
