# Supplementary material for: An update on the human and animal enteric pathogen Clostridium perfringens
Source: Emerg Microbes Infect. 2018 Aug 6;7:141. doi: 10.1038/s41426-018-0144-8 (PMC6079034; doi:10.1038/s41426-018-0144-8)
Supplement: Supplementary file 1 — Supplementary Table S1 [file 41426_2018_144_MOESM1_ESM.pdf]

Supplementary Table S1: Updated summary of the pathogenicity mechanisms of the currently identified/ characterised *Clostridium perfringens* toxins [1].

		**Gene**	**Toxin name**	**Alternative name**	**Mechanism of pathogenicity**	**Refs**
**1**	Typing toxins [2]	*plc/cpa*	Phospholipase	α-toxin	Disruption of cell membrane	3
**2**		*cpb*	β-toxin	-	Pore-formation	4
**3**		*etx*	ε-toxin	-	Pore-formation	5
**4**		*iap*	ι-toxin component Ia	-	Cytoskeleton disruption	6
**5**		*ibp*	ι-toxin component Ib	-	Cytoskeleton disruption	6
**6**		*cpe*	Enterotoxin (CPE)	-	Pore-formation and tight-junction disintegration	6
**7**		*netB*	NetB	-	Pore-formation	7
**8**	Non-typing toxins	*cpb2*	β2 toxin	-	Pore-formation	8
**9**		*lam*	λ-toxin	-	Potent protease	9
**10**		*pfo/pfoA*	Perfringolysin O	θ-toxin	Pore-formation	10
**11**		*cpd*	δ-toxin	-	Pore-formation	11
**12**		*ccp*	Clostripain	-	Digestion of collagen	12
**13**		*colA*	Microbial collagenase	κ-toxin	Digestion of collagen	13
**14**		*nanI*	Sialidase	-	Mucolysis	12
**15**		*nanJ*	Exo-α-sialidase	-	Mucolysis	12
**16**		*nanH*	Neuraminidase	-	Mucolysis	14
**17**		*nagH*	Hyaluronidase	μ-toxin	Digestion of connective tissue	12
**18**		*tpeL*	Glucosylating toxin	-	Induction of apoptosis	15
**19**		*becA*	Binary Enterotoxin Component A	-	Pore-formation	16
**20**		*becB*	Binary Enterotoxin Component B	-	Pore-formation	16
**21**		*netE*	NetE	-	Pore-formation	17
**22**		*netF*	NetF	-	Pore-formation	18
**23**		*netG*	NetG	-	Pore-formation	17

References

1 Kiu, R., Caim, S., Alexander, S., Pachori, P. & Hall, L. J. Probing Genomic Aspects of the Multi-Host Pathogen *Clostridium perfringens* Reveals Significant Pangenome Diversity, and a Diverse Array of Virulence Factors. *Front Microbiol* **8**, 2485, doi:10.3389/fmicb.2017.02485 (2017).

2 Rood, J. I. *et al.* Expansion of the *Clostridium perfringens* toxin-based typing scheme. *Anaerobe* (2018).

3 Titball, R. W., Naylor, C. E. & Basak, A. K. The *Clostridium perfringens* alpha-toxin. *Anaerobe* **5**, 51-64, doi:10.1006/anae.1999.0191 (1999).

4 Theoret, J. R., Uzal, F. A. & McClane, B. A. Identification and characterization of *Clostridium perfringens* beta toxin variants with differing trypsin sensitivity and in vitro cytotoxicity activity. *Infect. Immun.* **83**, 1477-1486, doi:10.1128/IAI.02864-14 (2015).

5 Nagahama, M. *et al.* Cellular vacuolation induced by *Clostridium perfringens* epsilon-toxin. *FEBS J.* **278**, 3395-3407, doi:10.1111/j.1742-4658.2011.08263.x (2011).

6 Miyamoto, K. *et al.* Identification of novel *Clostridium perfringens* type E strains that carry an iota toxin plasmid with a functional enterotoxin gene. *PLoS One* **6**, e20376, doi:10.1371/journal.pone.0020376 (2011).

7 Fernandes da Costa, S. P. *et al.* Identification of a key residue for oligomerisation and pore-formation of *Clostridium perfringens* NetB. *Toxins (Basel)* **6**, 1049-1061, doi:10.3390/toxins6031049 (2014).

8 Gibert, M., Jolivet-Reynaud, C. & Popoff, M. R. Beta2 toxin, a novel toxin produced by *Clostridium perfringens*. *Gene* **203**, 65-73 (1997).

9 Jin, F. *et al.* Purification, characterization, and primary structure of *Clostridium perfringens* lambda-toxin, a thermolysin-like metalloprotease. *Infect. Immun.* **64**, 230-237 (1996).

10 Ohno-Iwashita, Y., Iwamoto, M., Mitsui, K., Kawasaki, H. & Ando, S. Cold-labile hemolysin produced by limited proteolysis of theta-toxin from *Clostridium perfringens*. *Biochemistry* **25**, 6048-6053 (1986).

11 Manich, M. *et al. Clostridium perfringens* delta toxin is sequence related to beta toxin, NetB, and *Staphylococcus* pore-forming toxins, but shows functional differences. *PLoS One* **3**, e3764, doi:10.1371/journal.pone.0003764 (2008).

12 Shimizu, T. *et al.* Complete genome sequence of *Clostridium perfringens*, an anaerobic flesh-eater. *Proc Natl Acad Sci U S A* **99**, 996-1001, doi:10.1073/pnas.022493799 (2002).

13 Matsushita, O., Yoshihara, K., Katayama, S., Minami, J. & Okabe, A. Purification and characterization of *Clostridium perfringens* 120-kilodalton collagenase and nucleotide sequence of the corresponding gene. *J. Bacteriol.* **176**, 149-156 (1994).

14 Roggentin, P., Rothe, B., Lottspeich, F. & Schauer, R. Cloning and sequencing of a *Clostridium perfringens* sialidase gene. *FEBS Lett.* **238**, 31-34 (1988).

15 Jiang, Y. F., Kulkarni, R. R., Parreira, V. R. & Prescott, J. F. Immunization of Broiler Chickens Against *Clostridium perfringens*-Induced Necrotic Enteritis Using Purified Recombinant Immunogenic Proteins. *Avian Dis.* **53**, 409-415 (2009).

16 Yonogi, S. *et al.* BEC, a novel enterotoxin of *Clostridium perfringens* found in human clinical isolates from acute gastroenteritis outbreaks. *Infect. Immun.* **82**, 2390-2399, doi:10.1128/IAI.01759-14 (2014).
17 Mehdizadeh Gohari, I. *et al.* A novel pore-forming toxin in type A *Clostridium perfringens* is associated with both fatal canine hemorrhagic gastroenteritis and fatal foal necrotizing enterocolitis. *PLoS One* **10**, e0122684, doi:10.1371/journal.pone.0122684 (2015).
18 Gohari, I. M. *et al.* Plasmid Characterization and Chromosome Analysis of Two netF+ *Clostridium perfringens* Isolates Associated with Foal and Canine Necrotizing Enteritis. *PLoS One* **11**, e0148344, doi:10.1371/journal.pone.0148344 (2016).
