# Supplementary material for: An update on the human and animal enteric pathogen Clostridium perfringens
Source: Emerg Microbes Infect. 2018 Aug 6;7:141. doi: 10.1038/s41426-018-0144-8 (PMC6079034; doi:10.1038/s41426-018-0144-8)
Supplement: Supplementary file 2 — Supplementary Table S2 [file 41426_2018_144_MOESM2_ESM.pdf]

Supplementary Table S2: A summary of *Clostridium perfringens* antimicrobial resistance (both pheno- and genotypic) based on available literature. *High-prevalence antibiotic resistance reported.

**Antibiotic class**	**Inhibitory mechanism**	**High susceptibility**	**Moderate resistance**	**High resistance**	**Refs**
Penicillin	Cell wall synthesis	Penicillin G<br>Amoxicillin	Ampicillin		1-6
Glycopeptides	Cell wall synthesis	Vancomycin<br>Daptomycin			3,7
Imidazoles	Nucleic acid synthesis	Metronidazole			3,4
Cephalosporins	Cell wall synthesis	Cefotaxime	Ceftazidime<br>Ceftriaxone<br>Ceftiofur		3,5,6
Amphenicols	Protein synthesis	Chloramphenicol			1,3,4
Carbapenems	Cell wall synthesis		Imipenem		4
Tetracyclines	Protein synthesis			Tetracycline*<br>Minocycline<br>Doxycycline	1,5,8-11
Lincosamides	Protein synthesis			Lincomycin*<br>Clindamycin*	4,8,12
Macrolides	Protein synthesis			Erythromycin*<br>Tylosin	1,2,8,9,12
Quinolones	Nucleic acid synthesis			Norfloxacin<br>Enrofloxacin<br>Nalidixic acid	3
Aminoglycosides	Protein synthesis			Gentamycin<br>Streptomycin	2,6
Polypeptides	Cell wall synthesis			Bacitracin<br>Colistin	2,6

References

1 Rood, J. I., Maher, E. A., Somers, E. B., Campos, E. & Duncan, C. L. Isolation and characterization of multiply antibiotic-resistant *Clostridium perfringens* strains from porcine feces. *Antimicrob. Agents Chemother.* **13**, 871-880 (1978).

2 Osman, K. M. & Elhariri, M. Antibiotic resistance of *Clostridium perfringens* isolates from broiler chickens in Egypt. *Rev Sci Tech* **32**, 841-850 (2013).

3 Kouassi, K. A., Dadie, A. T., N'Guessan, K. F., Dje, K. M. & Loukou, Y. G. *Clostridium perfringens* and *Clostridium difficile* in cooked beef sold in Cote d'Ivoire and their antimicrobial susceptibility. *Anaerobe* **28**, 90-94, doi:10.1016/j.anaerobe.2014.05.012 (2014).

4 Akhi, M. T. *et al.* Antibiotic Sensitivity of *Clostridium perfringens* Isolated From Faeces in Tabriz, Iran. *Jundishapur J Microbiol* **8**, e20863, doi:10.5812/jjm.20863v2 (2015).

5 Yadav, J. P. *et al.* Molecular characterization and antimicrobial resistance profile of *Clostridium perfringens* type A isolates from humans, animals, fish and their environment. *Anaerobe* **47**, 120-124, doi:10.1016/j.anaerobe.2017.05.009 (2017).

6 Park, J. Y. *et al.* Characterization of *Clostridium perfringens* isolates obtained from 2010 to 2012 from chickens with necrotic enteritis in Korea. *Poultry Science* **94**, 1158-1164, doi:10.3382/ps/pev037 (2015).

7 Tyrrell, K. L. *et al.* In vitro activities of daptomycin, vancomycin, and penicillin against *Clostridium difficile*, *C. perfringens*, *Finegoldia magna*, and *Propionibacterium acnes*. *Antimicrobial Agents and Chemotherapy* **50**, 2728-2731, doi:10.1128/Aac.00357-06 (2006).

8 Slavic, D. *et al.* Antimicrobial susceptibility of *Clostridium perfringens* isolates of bovine, chicken, porcine, and turkey origin from Ontario. *Can J Vet Res* **75**, 89-97 (2011).

9 Soge, O. O., Tivoli, L. D., Meschke, J. S. & Roberts, M. C. A conjugative macrolide resistance gene, mef(A), in environmental *Clostridium perfringens* carrying multiple macrolide and/or tetracycline resistance genes. *J. Appl. Microbiol.* **106**, 34-40, doi:10.1111/j.1365-2672.2008.03960.x (2009).

10 Li, C., Yan, X. & Lillehoj, H. S. Complete Genome Sequence of *Clostridium perfringens* LLY_N11, a Necrotic Enteritis-Inducing Strain Isolated from a Healthy Chicken Intestine. *Genome Announc* **5**, doi:10.1128/genomeA.01225-17 (2017).

11 Kiu, R., Caim, S., Alexander, S., Pachori, P. & Hall, L. J. Probing Genomic Aspects of the Multi-Host Pathogen *Clostridium perfringens* Reveals Significant Pangenome Diversity, and a Diverse Array of Virulence Factors. *Front Microbiol* **8**, 2485, doi:10.3389/fmicb.2017.02485 (2017).

12 Ngamwongsatit, B. *et al.* Multidrug resistance in *Clostridium perfringens* isolated from diarrheal neonatal piglets in Thailand. *Anaerobe* **38**, 88-93, doi:10.1016/j.anaerobe.2015.12.012 (2016).
