# Supplementary material for: An update on the human and animal enteric pathogen Clostridium perfringens
Source: Emerg Microbes Infect. 2018 Aug 6;7:141. doi: 10.1038/s41426-018-0144-8 (PMC6079034; doi:10.1038/s41426-018-0144-8)
Supplement: Supplementary file 3 — Supplementary Table S3 [file 41426_2018_144_MOESM3_ESM.pdf]

Supplementary Table S3: *Clostridium perfringens*-associated intestinal diseases in both animals and humans.

Host species	Disease	Affected groups	Clinical symptoms	Associated toxinotypes/toxins	Refs
Cow	Bovine necrotic enteritis	Neonatal calves	Distended colon; mucosal necrosis	Type A/β2-toxin, perfringolysin O	1,2
Chicken	Poultry necrotic enteritis	Neonatal chicks 2-5 weeks post-hatching	Gaseous lesions; mucosal necrosis; distended intestines	Type A, C, G/β-toxin, NetB, TpeL	3
Pig	Swine enterocolitis	1-7 days old neonatal piglets	Severe diarrhoea, necrotic mucosa, villus atrophy	Type A, C/β2-toxin	4
Dog	Canine gastroenteritis	n/a	Haemorrhagic/ necrotic intestines	Type A, F/enterotoxin CPE, β2-toxin	5
Horse	Equine necrotising enterocolitis	1-14 days old neonatal foals	Bloody diarrhoea; haemorrhagic and necrotic intestines	Type A, F/enterotoxin CPE, β2-toxin, NetE, NetF & NetG	5
Human	Acute watery diarrhoea (food poisoning)	Both children and adults	8-14h after food ingestion: intestinal cramp, watery diarrhoea without vomiting (self-limiting: 12-24h)	Type F/enterotoxin CPE	6
Human	Non-foodborne diarrhoea (antibiotic-associated diarrhoea and sporadic diarrhoea)	Older adults (>60 years old)	Abdominal pain and diarrhoea (prolonged duration >3 days to several weeks), often accompanied by blood in the faeces.	Type F/enterotoxin CPE	7
Human	Enteritis necroticans (Pigbel)	Children/ malnourished adults	Intestinal gangrene (small intestine as key infection site)	Type C/β-toxin	8
Human	Pre-term necrotising enterocolitis	1-14 days old neonatal pre-term infants	Distended abdomen; pneumatosis intestinalis (gas cysts formation); intestinal necrosis	Type A/β2-toxin	9,10

References

1. Nowell, V. J. *et al.* Genome sequencing and analysis of a type A *Clostridium perfringens* isolate from a case of bovine clostridial abomasitis. *PLoS One* **7**, e32271, doi:10.1371/journal.pone.0032271 (2012).
2. Verherstraeten, S. *et al.* The synergistics necrohemorrhagic action of *Clostridium perfringens* perfringolysin and alpha toxin in the bovine intestine and against bovine endothelial cells. *Vet. Res.* **44**, 45 (2013).
3. Nakano, V. *et al.* Multilocus sequence typing analyses of *Clostridium perfringens* type A strains harboring tpeL and netB genes. *Anaerobe* **44**, 99-105, doi:10.1016/j.anaerobe.2017.02.017 (2017).
4. Chan, G. *et al.* The epidemiology of *Clostridium perfringens* type A on Ontario swine farms, with special reference to cpb2-positive isolates. *BMC Vet Res* **8**, 156, doi:10.1186/1746-6148-8-156 (2012).
5. Gohari, I. M. *et al.* Plasmid Characterization and Chromosome Analysis of Two netF+ *Clostridium perfringens* Isolates Associated with Foal and Canine Necrotizing Enteritis. *PLoS One* **11**, e0148344, doi:10.1371/journal.pone.0148344 (2016).
6. Johansson, A. *et al.* Genetic diversity of *Clostridium perfringens* type A isolates from animals, food poisoning outbreaks and sludge. *BMC Microbiol.* **6**, 47, doi:10.1186/1471-2180-6-47 (2006).
7. Larson, H. E. & Borriello, S. P. Infectious diarrhea due to *Clostridium perfringens*. *J. Infect. Dis.* **157**, 390-391 (1988).
8. Murrel, T. G. C. Pigbel in Papua New Guinea: An ancient disease rediscovered. *Int. J. Epidemiol.* **12**, 211-214 (1983).
9. Sim, K. *et al.* Dysbiosis anticipating necrotizing enterocolitis in very premature infants. *Clin. Infect. Dis.* **60**, 389-397, doi:10.1093/cid/ciu822 (2015).
10. Heida, F. H. *et al.* A necrotizing enterocolitis-associated gut microbiota is present in the meconium: results of a prospective study. *Clin. Infect. Dis.*, doi:10.1093/cid/ciw016 (2016).
