# Supplementary material for: An update on the human and animal enteric pathogen Clostridium perfringens
Source: Emerg Microbes Infect. 2018 Aug 6;7:141. doi: 10.1038/s41426-018-0144-8 (PMC6079034; doi:10.1038/s41426-018-0144-8)
Supplement: Supplementary file 4 — Supplementary Table S4 [file 41426_2018_144_MOESM4_ESM.pdf]

Supplementary Table S4: Studies reporting clinical cases (with key details) of *Clostridium perfringens*-associated Necrotising Enterocolitis (NEC) since 1970s.

Reporting year	Major findings	Pathology/ disease presentations	Identification technique/ strains identified	Refs
1976	*C. perfringens* isolated from 6 out of 7 NEC patients	Pneumatosis intestinalis	Culturing/ *C. perfringens* type A	1
1978	*C. perfringens* detected in 3 of 4 deceased NEC patients	Severe pneumatosis intestinalis	Culturing/ *C. perfringens*	2
1984	*C. perfringens* isolated from 5 NEC patients, 2 died; *C. perfringens* being the most found pathogen in 50 NEC patients in a 4-year cohort of 375 babies.	Pneumatosis intestinalis, portal venous gas, sepsis	Culturing/ *C. perfringens*	3
1984	*C. perfringens* detected in 2 NEC patients	n/a	Culturing/ *C. perfringens*	4
1985	*C. perfringens* detected in 10 NEC patients with 78% fatal outcome	n/a	Culturing/ *C. perfringens*	5
1985	*C. perfringens* detected in 9 NEC patients with 78% fatal outcome	Intestinal gangrene and extensive pneumatosis intestinalis	Culturing/ *C. perfringens*	6
1990	*C. perfringens* was more abundant in 1 out of 4 NEC infants	n/a	Culturing/ *C. perfringens*	7
2004	Case-control study of 12 pre-term infants. 3 *C.-perfringens*-positive infants developed NEC later (100%) and eventually died (100%)	n/a	16S rRNA PCR/ *C. perfringens*	8
2008	Case-control study of 9 NEC patients associated with *C. perfringens*. Outcomes compared with non-*C. perfringens*-NEC (n=32): higher mortality (44% vs 7%) and high percentage of portal venous gas (78% vs 25%)	Abdominal distention; bloody stools; pneumatosis intestinalis; portal venous gas	Culturing; multiplex-PCR toxinotyping/ *C. perfringens* type A	9

**Reporting year**	**Major findings**	**Pathology/ disease presentations**	**Identification technique/ strains identified**	**Refs**
2009	*C. perfringens* isolated from necrotic colon wall of 1 NEC pre-term patient	Abdominal distention; necrotic intestine; pneumatosis intestinalis; multiple perforation; haemolysis	Culturing; Nagler reaction on egg yolk agar	10
2010	*C. perfringens* isolated from 3 NEC patients (faecal samples)	n/a	Culturing; 16S rRNA PCR; multiplex-PCR toxinotyping/ *C. perfringens* type A	11
2010	*C. perfringens* detected from 3 NEC patients (peritoneal fluid samples) who eventually died from multisystem organ failure	Abdominal distention; pneumatosis intestinalis; extensive bowel necrosis	Intra-hospital culturing	12
2015	A 2-year cohort study of 369 pre-term infants. Overabundant *C. perfringens* were detected in 4 NEC (Bell 2/3) patients before disease onset (in a total of 12 NEC patients) using NGS technology	n/a	16S rRNA metagenomics (V3-V5 regions); culturing and MALDI-TOF; multiplex-PCR toxinotyping/ *C. perfringens* type A, 3 isolates harboured β2-toxin	13
2016	A case-control study of 3 NEC patients. *C. perfringens* were found to be significantly more abundant from meconium to NEC onset.	Pneumatosis intestinalis and/or portal venous gas	16S rRNA metagenomics (V3-V4 regions)	14

References

1 Pedersen, P. V., Hansen, F. H., Halveg, A. B. & Christiansen, E. D. Necrotising enterocolitis of the newborn--is it gas-gangrene of the bowel? *Lancet* **2**, 715-716 (1976).

2 Kosloske, A. M., Ulrich, J. A. & Hoffman, H. Fulminant necrotising enterocolitis associated with clostridia. *Lancet* **312**, 1014-1016 (1978).

3 Yu, V. Y., Joseph, R., Bajuk, B., Orgill, A. & Astbury, J. Necrotizing enterocolitis in very low birthweight infants: a four-year experience. *Aust Paediatr J* **20**, 29-33 (1984).

4 Warren, S., Schreiber, J. R. & Epstein, M. F. Necrotizing enterocolitis and hemolysis associated with *Clostridium perfringens*. *American journal of diseases of children (1960)* **138**, 686-688 (1984).

5 Blakey, J. L. *et al.* Development of gut colonisation in pre-term neonates. *J. Med. Microbiol.* **15**, 519-529, doi:10.1099/00222615-15-4-519 (1982).

6 Kosloske, A. M., Ball Jr, W. S., Umland, E. & Skipper, B. Clostridial necrotizing enterocolitis. *J. Pediatr. Surg.* **20**, 155-159 (1985).

7 Hoy, C. *et al.* Quantitative changes in faecal microflora preceding necrotising enterocolitis in premature neonates. *Arch. Dis. Child.* **65**, 1057-1059 (1990).

8 De La Cochetiere, M. F. *et al.* Early intestinal bacterial colonization and necrotizing enterocolitis in premature infants: The putative role of *Clostridium*. *Pediatric Research* **56**, 366-370 (2004).

9 Dittmar, E. *et al.* Necrotizing enterocolitis of the neonate with *Clostridium perfringens*: Diagnosis, clinical course, and role of alpha toxin. *Eur. J. Pediatr.* **167**, 891-895 (2008).

10 Hanke, C. A. *et al. Clostridium perfringens* intestinal gas gangrene in a preterm newborn. *Eur J Pediatr Surg* **19**, 257-259, doi:10.1055/s-2008-1038958 (2009).

11 Kotsanas, D. *et al.* Novel use of tryptose sulfite cycloserine egg yolk agar for isolation of *Clostridium perfringens* during an outbreak of necrotizing enterocolitis in a neonatal unit. *J. Clin. Microbiol.* **48**, 4263-4265 (2010).

12 Schlapbach, L. J., Ahrens, O., Klimek, P., Berger, S. & Kessler, U. *Clostridium perfringens* and necrotizing enterocolitis. *J Pediatr* **157**, 175, doi:10.1016/j.jpeds.2010.03.027 (2010).

13 Sim, K. *et al.* Dysbiosis anticipating necrotizing enterocolitis in very premature infants. *Clin. Infect. Dis.* **60**, 389-397, doi:10.1093/cid/ciu822 (2015).

14 Heida, F. H. *et al.* A necrotizing enterocolitis-associated gut microbiota is present in the meconium: results of a prospective study. *Clin. Infect. Dis.*, doi:10.1093/cid/ciw016 (2016).
